# Hybrid Feature Extraction for Detection of Degree of Motor Fluctuation Severity in Parkinson’s Disease Patients

**DOI:** 10.3390/e21020137

**Published:** 2019-02-01

**Authors:** Murtadha D. Hssayeni, Joohi Jimenez-Shahed, Behnaz Ghoraani

**Affiliations:** 1 Department of Computer and Electrical Engineering and Computer Science, Florida Atlantic University, Boca Raton, FL 33431, USA; 2 Department of Neurology, Baylor College of Medicine, Houston, TX 77030, USA

**Keywords:** Parkinson’s disease, wearable sensors, machine learning, hybrid feature extraction, random forest decision trees, self-organizing tree map, motor fluctuation

## Abstract

The success of medication adjustment in Parkinson’s disease (PD) patients with motor fluctuation relies on the knowledge about their fluctuation severity. However, because of the temporal and spatial variability in motor fluctuations, a single clinical examination often fails to capture the spectrum of motor impairment experienced in routine daily life. In this study, we developed an algorithm to estimate the degree of motor fluctuation severity from two wearable sensors’ data during subjects’ free body movements. Specifically, we developed a new hybrid feature extraction method to represent the longitudinal changes of motor function from the sensor data. Next, we developed a classification model based on random forest to learn the changes in the patterns of the sensor data as the severity of the motor function changes. We evaluated our algorithm using data from 24 subjects with idiopathic PD as they performed a variety of daily routine activities. A leave-one-subject-out assessment of the algorithm resulted in 83.33% accuracy, indicating that our approach holds a great promise to passively detect degree of motor fluctuation severity from continuous monitoring of an individual’s free body movements. Such a sensor-based assessment system and algorithm combination could provide the objective and comprehensive information about the fluctuation severity that can be used by the treating physician to effectively adjust therapy for PD patients with troublesome motor fluctuation.

## 1. Introduction

Parkinson’s disease (PD) is a chronic, progressive neurological disorder affecting 1–1.5 million Americans, which is predicted to double by 2040 [1]. PD leads to disabling motor features including tremor, bradykinesia/akinesia (reduced speed and quantity of spontaneous movement), and gait/balance impairment leading to falls, as well as non-motor symptoms such as cognitive impairment and sleep and speech disorders. PD has rapidly growing societal and economic impacts with estimated costs of about $24,000 per patient, resulting in total cost of over $20 billion per year [2].

Motor fluctuations between mobile phases, known as the medication “ON” states with maximum benefit from medication, and akinetic phases, referred to as “OFF” states when the effects have worn off, are one of the most prevalent complications of patients with PD. These medication ON and OFF fluctuations occur in 50% of patients within 3 to 5 years of diagnosis and 80% of patients after 10 years [3]. The onset of motor fluctuations marks a crucial point in the management of PD as it involves the need for ongoing therapy adjustments, such as medication frequency and dosage or deep brain stimulation parameters.

One of the main contributing factors to a successful therapeutic adjustment is recognition of the degree of motor fluctuation severity between medication ON and OFF states [4], which is obtained via brief clinical examinations or depends on appropriate history-taking and subject report. A more detailed but time-consuming clinical evaluation can be performed, where a neurologist evaluates a series of motor tasks in the patient’s ON and OFF states, and scores (scale 0 to 4) the patient’s motor function according to the guidelines of Unified Parkinson’s Disease Rating Scale (UPDRS) [5]. The UPDRS is composed of four subscales (Part IIV) where Part III measures severity of motor complications and is performed by clinical examinations. The measured or estimated Average Change between the ON and OFF states using either the UPDRS Part III scores (AC-UPDRS III) or clinical judgment provides a measure of the degree of motor fluctuation severity. Such knowledge about the degree of motor fluctuation severity (i.e., being minimal, mild, moderate, or severe) can be used by the neurologist to make changes to medication dose, frequency, and/or type as needed. However, the assessment is not entirely objective and has shown to be error prone due to personal judgments and experience and it is based on a discrete scaling, which complicates and limits statistical analysis to detect subtle changes during the disease progression [6,7]. Moreover, the fluctuation status can change dramatically over the course of a few hours and may involve different body segments at different times. This temporal and spatial variability in motor fluctuation manifestations makes it challenging to capture the spectrum of motor impairment experienced in routine daily life from a single channel examination.

Technology-based assessments of a patient’s response to therapy, using wearable sensors, holds great promise in providing objective clinically actionable information that can be used by the treating physician to effectively adjust therapy for PD patients with troublesome motor fluctuation [8]. Recently, technology-based assessments have been developed for automatic assessment of severity of motor symptoms [9,10,11,12,13]. However, they generate abstract indices about motor impairments (i.e., tremor and bradykinesia), which have not been associated with the degree of motor fluctuation severity that is required for the treating physician to effectively adjust therapy. Other approaches attempt to estimate UPDRS III scores from each time point based on some symptom-based features (e.g., spectral power in 4–6 Hz frequencies for tremor and 1–4 Hz frequencies for bradykinesia) [14,15,16,17,18,19,20,21] or deep learning algorithms [22,23]. Such approaches could be useful in detection of PD from healthy subjects. However, their primary downfall for PD therapy adjustment is that they still require the information about the medication ON and OFF states to translate the detected UPDRS III scores into clinically actionable information (i.e., ACUPDRS III score). More importantly, they are non-passive, meaning that they require subjects’ active engagement to perform specific tasks (tapping on the phone, uttering specific sounds, drawing, and walking) with the aim of eliciting PD symptoms. Hence, these methods can provide only an isolated “snapshot” measurement of the severity only under optimized and controlled conditions, and hence may be of limited value [6].

In this study, we describe a new approach to estimate a subject’s degree of motor fluctuation severity. Our approach is fundamentally different from the existing approaches in three main aspects: (1) Instead of estimating PD symptoms’ severity or UPDRS III scores as in previous studies, it estimates the clinically actionable feature, ACUPDRS III which could potentially be used to improve the effectiveness of the PD treatment management system; (2) Instead of requiring the subjects to perform specific tasks, it passively detects degree of motor fluctuation severity from continuous monitoring of an individual’s motor function during daily, free-living condition; and (3) Instead of using symptom-based features from each time point, it uses features from longitudinal changes of motor function by developing the first hybrid feature extraction method for PD data analysis. We demonstrate that the information from two wearable sensors placed on the upper and lower extremity of PD subjects is sufficient for our algorithm to predict the degree of motor fluctuation severity assessed from clinical examinations (i.e., ACUPDRS III degrees of severity).

## 2. Materials and Methods

### 2.1. Participants and Data Collection

A total of 24 subjects with idiopathic Parkinson’s disease (disease duration of 3.5 to 17 years; mean 9.9 years), taking Levodopa with motor fluctuations based on a neurologist’s judgment and a Hoehn and Yahr scale [24] range of 2 to 4. The subjects did not have a known diagnosis of dementia. The study was collected under the protocol in Refs. [25,26] and approved by the institutional review board. All participants provided written informed consent before the study. See Table 1 for patients’ characteristics. Two wearable sensors (Kinesia motion sensor units, Great Lakes NeuroTechnologies Inc.; Cleveland, OH, USA) were mounted on the back of the most PD-affected wrist and the front of the most PD-affected ankle, and their gyroscope data were later extracted to the MATLAB program for further analysis.

The participants were asked to stop their medication the night before the scheduled examination so they will start the experiment in their medication OFF state. The subjects performed a variety of daily living activities in a homelike setting while engaging in unconstrained activities (such as resting, walking, drinking, dressing, hair brushing, unpacking groceries, and cutting food) before taking their prescribed dose of Levodopa and repeated the activities 24 times during 24 h after taking Levodopa. Concurrently, the clinical examinations were performed by a neurologist to record patients’ medication ON and OFF states. Several rounds of UPDRS III assessment were performed for each subject: at the beginning of every hour of the experiment for 15 subjects and at the beginning and end of the experiment for nine of the participants. The OFF state UPDRS III scores ranged from 7 to 60 with an average of 29.7 and standard deviation (SD) of 12.3 (see Table 1). Out of 24 subjects, four did not convert to their ON state, so ACUPDRS III score was reported as inconclusive (INC) for those four subjects. The ACUPDRS III score of the remaining 20 subjects was calculated as the difference between the average OFF and ON UPDRS III scores. The degree of motor fluctuation severity is then classified into four categories: minimal (ACUPDRS III ≤ 5), mild (5 ≤ 5), mild (5 < ACUPDRS III ≤ 10), moderate (10 < ACUPDRS III ≤ 15), and severe (15 < ACUPDRS III) changes in motor fluctuation. This resulted in four subjects in each of the minimal, mild, and moderate motor fluctuation categories and eight subjects in the severe motor fluctuation category.

### 2.2. Classification of Motor Fluctuation Severity: Problem Formulation

**Figure 1:**
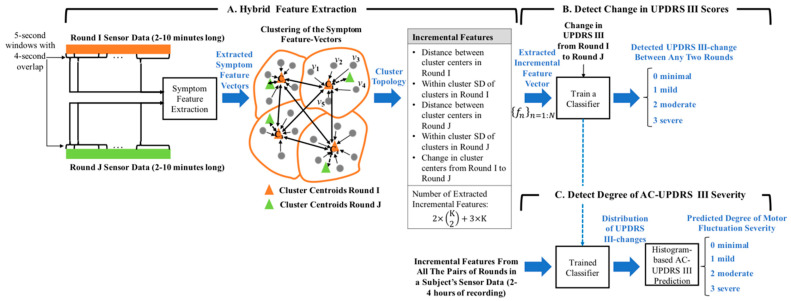
Graph representing the developed algorithm to predict degree of motor fluctuation severity. The algorithm consists of three stages: (**A**) hybrid feature extraction to extract incremental features that represent longitudinal changes in any two sensor-signal segments (referred to rounds); (**B**) A classifier to detect distribution of changes in UPDRS III scores between different rounds in a subject’s data; and (**C**) A decision-making system to assign an ACUPDRS III score (0 to 3) according to the UPDRS III change distribution and predict degree of motor fluctuation severity.

We developed an algorithm (see Figure 1) to estimate motor fluctuation severity (0: minimal, 1: mild, 2: moderate, and 3: severe) from two wearable sensors’ data from subjects’ complex movements as in their home environment, without the need for the subjects to perform specific tasks. In general, the algorithm estimates the degree of change in the ACUPDRS III scores by a feature extraction/machine leaning approach (Figure 1A,B), followed by a histogram-based decision-making process (Figure 1C). The former develops a *classifier* that estimates the degree of change in the UPDRS III scores between any two segments of the data with a duration of 210 min (referred to rounds here after). The latter estimates the ACUPDRS III degree of severity of a subject from the histogram of the degree of change in the UPDRS III scores that is obtained from the classification results of the *trained classifier* on all the possible pairs of rounds in the subject’s 24 h of recording. Please note that the *classifier* makes an intermediate estimation of the degree of change in the UPDRS III scores between two rounds, which is used in the histogram-based decision-making process to detect the clinically meaningful ACUPDRS III score of 0 to 3 and estimate a subject’s degree of motor fluctuation severity being minimal, mild, moderate, or severe.

The feature extraction aspect of the algorithm is based on a hybrid feature extraction (Figure 1A), which represents the longitudinal changes between any two rounds of data as one feature vector. The method is composed of a symptom-based and an incremental feature extraction. The symptom-based feature vectors are extracted from windowed segments (5 s windows with 4 s overlaps) of every round (duration of 210 min) to represent the manifestation of the tremor and bradykinesia symptoms within a round (Figure 1C). One incremental feature vector is extracted to represent the longitudinal changes in the structure of symptom-based feature vectors (i.e., differences in the PD symptom manifestations) between any two rounds of data. This incremental feature vector is then fed to the classifier to model and estimate the degree of change in the UPDRS III scores between the two rounds. The details of each part are described next.

### 2.3. Data Processing and Arrangement

**Figure 2:**
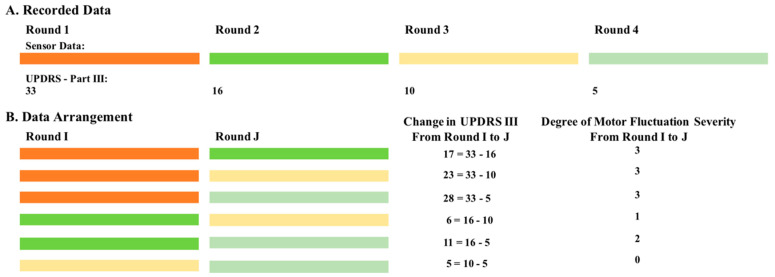
A diagram demonstrating the data arrangement into pairs of rounds in case of subject #1. (**A**) Four rounds of data along with their associated UPDRS III scores are shown. (**B**) All the possible pairs of rounds along with their associated change in UPDRS III scores and degree of motor fluctuation severity (0: minimal; 1: mild; 2: moderate; and 3 severe).

The sensor signals were preprocessed by applying a bandpass FIR filter with a pass frequency between 0.5 Hz to 15 Hz on the three axes of the recorded signal from each sensor to eliminate low and high-frequency noises. Next, from each subject’s sensor data, we selected the data rounds for which a clinical assessment was concurrently performed and the associated UPDRS III score was provided. We arranged this data into pairs of rounds and used them to train an algorithm that learns the changes in the patterns of the sensor data as the UPDRS III score changes from one round to another. This data arrangement was performed in the following two steps. In step 1, the collected data was divided into two to four rounds, where each round was defined by a UPDRS III score and its concurrently recorded sensor data (i.e., gyroscope signals). A symbolic diagram of this process in case of subject #1 is shown in Figure 2A. Four UPDRS III scores were recorded so four rounds of data with their associated UPDRS III scores (33, 16, 10, and 5 for Rounds 1 to 4, respectively) were selected for this subject. For this particular subject, AC_UPDRS III is 22.67 (= average {33} − average {16, 10, 5}), which is the difference between the average OFF state UPDRS III score (Round 1) and the average ON state UPDRS III score (Rounds 24). Given this strategy, a total of 91 rounds of data (duration of 4.11 ± 1.92 min per each round) were selected from all the 24 subjects.

In step 2, the data rounds for each subject were arranged into pairs with all the possible combinations. These pairs were then used to train a classifier that models the degree of change in the UPDRS III scores between any two rounds of data. As shown in Figure 2B, six possible pairs were constructed for subject #1 with four rounds of data. The data arrangement was repeated for all the subjects and resulted in a total of 130 pairs of rounds. Finally, the degree of motor fluctuation severity of each pair of rounds (e.g., Rounds I and J) was assigned to minimal, mild, moderate, and severe depending on the change in their UPDRS III score from Round I to Round J using the same cutoffs as was performed on ACUPDRS III (see Section 2.1). Out of 130 pairs of rounds, 66 were assigned to minimal motor fluctuation. The numbers of mild, moderate, and severe were 20, 17, and 27, respectively.

### 2.4. Hybrid Feature Extraction

We developed a feature extraction method to quantify the data signatures that define changes in the sensor data with different UPDRS III scores. The algorithm presents a new hybrid feature extraction approach that provides the incremental changes of the symptom-based features. As shown in Figure 1A, the proposed hybrid feature extraction algorithm consists of two stages: symptom-based feature extraction and incremental feature extraction. The input of the hybrid feature extraction consists of the sensor data of a pair of rounds (e.g., Round I and Round J). It extracts $M_{I}$ symptom-based feature vectors ${\left\{v_{m}\right\}}_{m=1:M_{I}}$ from Round I and $M_{J}$ of those feature vectors ${\left\{v_{m}\right\}}_{m=1:M_{J}}$ from the Round J data. The numbers of symptom-based feature vectors (i.e., $M_{I}$ and $M_{J}$) depend on the duration of each round. The symptom-based feature vectors of the two rounds are then converted into one incremental feature vector f, which represents the longitudinal changes in the PD symptom manifestation between the two rounds.

#### 2.4.1. Symptom-Based Feature Extraction

The filtered sensor data was segmented into 5second windows with 4second overlaps [27]. We extracted 66 signal features from every segment. Table 2 lists the extracted features and the description of the extracted features can be found in Ref. [28]. All the features except for cross correlation XY, XZ, and YZ were extracted from each X, Y, and Z signal separately. The amplitude-based features were extracted from the time domain signal and the spectral-based features (the shaded rows in Table 2) were extracted from the Fourier domain. The symptom-based features were extracted such that they represent one or both main motor symptoms (i.e., bradykinesia and tremor). The extracted symptom-based feature vectors from a round are denoted as ${\left\{v_{m}\right\}}_{m=1:M}$, where feature vector $v_{m}\in {\mathbb{R}}^{66\times 1}$ represents every fivesec segment of the sensor data. M is the number of the feature vectors extracted from a round and depends on the duration of the round.

#### 2.4.2. Incremental Feature Extraction

We represented the incremental changes in the sensor data from Round I to Round J by quantifying the changes in the distribution of the symptom-based feature vectors $\mathrm{the}\;\mathrm{two}\;\mathrm{rounds}\;{\left\{v_{m}\right\}}_{m=1:M_{I}}\;\mathrm{and}\;{\left\{v_{m}\right\}}_{m=1:M_{J}}\;\mathrm{in}\;\mathrm{the}\;\mathrm{feature}\;\mathrm{domain}$. First, the symptom-based feature vectors of Round I were clustered into *K* clusters with $C_{k}^{I}$ being the centroid of cluster *k* with $m_{k}$ cluster members: ${\left\{v_{n}\right\}}_{n=1:m_{k}}$. The clustering process was repeated on the symptom-based feature vectors of Round J and *K* clusters with $C_{k}^{J}$ cluster centroids were identified. Next, the changes in the clusters’ topology from the two clustering phases were extracted as the incremental features. These features were selected such that they signify the changes in the data pattern from Round I to Round J. The derived clusters from Rounds I and J were sorted in a descending order of the Euclidean distance of the cluster nodes: $d_{C_{1}^{I}}>d_{C_{2}^{I}}>\cdots >d_{C_{K}^{I}}$ and $d_{C_{1}^{J}}>d_{C_{2}^{J}}>\cdots >d_{C_{K}^{J}}$. The incremental features were extracted as the distances between cluster centroids from Round I (Equation (1)), within cluster SD of each cluster from Round I (Equation (2)), the distances between cluster centroids from Round J (Equation (1) for Round J), within cluster SD of each cluster from Round J (Equation (2) for Round J), and changes in the cluster nodes from Round I to Round J (Equation (3)). A total of $N=2\left(\begin{array}{c} K\\ 2 \end{array}\right)+3K$ incremental features ${\left\{f_{n}\right\}}_{n=1:N}$ were extracted from every pair of rounds. For example, N=35 features were extracted in case of K=5.

(1) $$d_{C_{12}},\ldots ,\;d_{C_{1K}},\;d_{C_{23}},\ldots ,\;d_{C_{K-1K}}$$

where $d_{c_{kk+1}}$ is the Euclidean distance between $C_{k}$ and $C_{k+1}$ for k=1 to K.

(2) $$\sqrt{\frac{\sum_{n=1}^{m_{k}}d_{v_{n}C_{k}}}{m_{k}-1}}\;\;\mathrm{for}\;k=1,\ldots ,\;K$$

where $d_{v_{n}C_{k}}$ is the Euclidean distance between $v_{n}$ and its cluster center $C_{k}$

(3) $$d_{C_{k}^{I}C_{k}^{J}}\;=\;\mathrm{Euclidean}\;\mathrm{distance}\;\mathrm{between}\;C_{k}^{I}\;\mathrm{and}\;C_{k}^{J}\;\mathrm{for}\;k=1,\ldots ,\;K$$

#### 2.4.3. Self-Organizing Tree Map for Clustering

For clustering purposes, we used self-organizing tree map (SOTM) method to map the extracted symptom-based feature vectors from their high-dimensional space into their representative clusters [29]. SOTM is a tree structure neural network that creates the clusters according to the data dynamics as the data grows in time. The algorithm is suitable for our application since the appropriate number of clusters is not known beforehand. If we use a clustering method (e.g., K-means) that requires a prespecified value of K, then we need to decide on an appropriate number of clusters by performing several random inner splits on the training data to select the best K size for every cross-validation. Instead of this process, we took advantage of SOTM to select the most appropriate K size. The SOTM algorithm initializes a tree of clusters by adding a root node for the first random feature vector and then it grows the tree by adding new nodes for the feature vectors that show dissimilarity to the closet node. Node creation is controlled by a dynamic hierarchical control function H(r) that decays with each iteration r to explore different levels of similarity between each node’s feature vectors. The resolution of clustering is imposed by hierarchical function H(r) in Equation (4). The nodes are adjusted using a learning rate α(r). Initially, α(r) is set as 0.8 and exponentially decays with each iteration as shown in Equation (5). In this work, we used a global learning rate that is shared for all the nodes. The process of SOTM is listed in Algorithm 1.

(4) $$H\left(r\right)=H\left(0\right)\left(1-\exp(-\frac{r}{2N\;}\right))$$

(5) $$\alpha \left(r\right)=0.8\;\exp\left(-\frac{r}{\tau }\right)$$

H(0) is the sum of the ranges of all the features, N is the number of feature vectors, and τ is set as 0.1. We selected a fix value of K for the number of clusters at every cross-validation. First, we applied the SOTM algorithm on all the pairs of rounds in the training data, and then used the median number of clusters as fix K and used it as MaxCls in line 9 of Algorithm 1, and also removed the stopping criteria of r being equal to MaxItr. In this way, the same number of incremental features were extracted during every classifier training and testing of every cross-validation. The average number of clusters *K* for our dataset was 4.25±0.34.

**Algorithm 1** Self-Organizing Tree Map for Incremental Feature Extraction**Input:** Feature vectors: $\left\{f_{1},\ldots ,f_{N}\right\}$; maximum number of iterations: MaxItr; maximum number of clusters: MaxCls
Initialize the root node $C_{1}\left(1\right)$ with a randomly selected feature vector**repeat** {Initialize r = 1 and k = 1}Take another feature vector f(r) randomly and find the closet node $C_{k}^{*}\left(r\right)$ based on the minimum Euclidean distance $d_{k}^{*}$.Calculate H(r) using Equation (4).Calculate α(r) using Equation (5).**If**$d_{k}^{*}\leq \;H\left(r\right)$**then**update node $C_{k}^{*}\left(r\right)$ using Equation (6)
(6)$$C_{k}^{*}\left(r+1\right)=C_{k}^{*}\left(r\right)+\alpha \left(r\right)\left[f\left(r\right)-C_{k}^{*}\left(r\right)\right]$$**else** increment k and initialize the new node $C_{1}\left(k\right)$ with f(r)Increment r**until**r is equal to MaxItr or k is equal to MaxCls.
**Output:** Cluster centroids $C_{1},\;\ldots ,\;C_{K}$ and their associated feature vectors ${\left\{v_{n}\right\}}_{n=1:m_{k}}$.

### 2.5. Detect Change in UPDRS III Scores

We developed a classification model to learn the degree of change in UPDRS III scores from Round I to Round J using the sensor data from the two rounds in one pair. The classifier receives the incremental feature vector extracted from the two rounds and assigns a severity score (0: minor, 1: mild, 2: moderate, and 3: severe) that represents the degree of motor fluctuation severity as the subject transits from Round I to Round J. For classification purposes, we used a random forest (RF) classifier [30]. Instead of learning single decision trees, which tends to overfit, the RF algorithm uses an ensemble classifier approach that learns multiple weak decision trees (high variance but low bias) using a randomly selected subset of feature vectors and feature attributes. The RF classifier has received increasing attention in many applications due to its generalization ability and robustness to noise as well processing speed for high-dimensional data [31,32,33,34]. The algorithm grows the decision trees in the ensembles by drawing a subset of feature vectors through a bagging approach (i.e., feature vectors are replaced back). Normally, two thirds of the feature vectors are used to train the trees and the remaining feature vectors are used to estimate the classification error, referred to as out-of-bag classification error, in an internal cross-validation. Once T ensemble decision trees are trained, they are used to classify a new feature vector by combining the results of all the trees. For this purpose, the new feature vector is evaluated against all the decision trees in the ensemble and the category with the majority vote of all the decision trees is assigned to the feature vector. We assigned the maximum number of decision trees to T=100 in the RF algorithm.

**Analysis Framework:** In this analysis, we considered the data form all the 24 subjects. Our training set consisted of the feature vectors of all the subjects except one and the testing set consisted of the feature vectors of the remaining subject. For example, in case of subject #1 (with four rounds and six incremental feature vectors), RF was trained on 124 (=130−6) feature vectors and tested on the remaining six feature vectors of subject #1. This leave-one-subject-out cross-validation approach was repeated for all the subjects and the estimated degrees of change in UPDRS III score were obtained for all the pairs of rounds. The average detection accuracy was considered as the generalized performance for detecting degrees of change in UPDRS III scores between any two rounds of sensor data. Additionally, we investigated the performance of the algorithm when no clustering was performed. The approach details and analysis are provided in the Supplement Materials.

### 2.6. Detect Degree of ACUPDRS III Severity

We developed a histogram-based decision-making process to detect a subjects’ degree of motor fluctuation severity (ACUPDRS III: 0: minor, 1: mild, 2: moderate, and 3: severe) from the classification results of the trained RF classifier on the subject’s sensor data. We performed the following three steps. Step 1: A RF classifier was modeled using the sensor data of all the subjects except one subject. The sensor data of the remaining subject was then segmented into rounds with a duration of 3 min and the rounds were paired using the same method explained in Section 2.3. The trained RF classifier was applied to every pair of rounds of the remaining subject and predicted the degree of change in UPDRS III score (0 to 3) for each pair. Step 2: The predicted values were used to create a distribution of all the UPDRS III changes in the subject’s data. The UPDRS III change distribution was computed as the number of occurrence of pairs of rounds that were predicted as each severity score of 0 to 3. For example, the UPDRS III change distribution for subject one was $d_{0}=1$, $d_{1}=0$, $d_{2}=1$, and $d_{3}=4$ corresponding to degree of change in UPDRS III score of 0, 1, 2, and 3, respectively. Step 3: We used Algorithm 2 to calculate the percentage of data with different degrees of ACUPDRS III severity based on the UPDRS III change distribution and detect the subject’s degree of motor fluctuation severity (*S*). As described in Algorithm 2, the ratio of the total number of the three degrees of UPDRS III severity of 1 to 3 (sum of $d_{1}$ to $d_{3}$) to the number of UPDRS III changes of 0 ($d_{0}$) was used to make the following decisions. If this ratio was greater than 0.3, the algorithm excluded the number of UPDRS III changes of 0 ($d_{0}$) in the ACUPDRS III percentage calculation. This condition was designed to eliminate the effect of the time durations that the subject did not experience any motor fluctuations (medication fully OFF or ON states) on the final decision. If the ratio was less than or equal to 0.2 (i.e., the degree of severity of 0 was in majority), the number of $d_{1}$ to $d_{3}$ were excluded from the analysis, and the algorithm decided that the degree of severity was 0. Finally, in the case that the ratio was between 0.2 and 0.3, the algorithm resulted in INC meaning that there was not sufficient information for the algorithm to decide about the degree of ACUPDRS III severity for the given duration of the data.

**Analysis Framework:** The three steps were repeated in a leave-one-subject-out cross-validation approach for all the subjects. The predicted degree of motor fluctuation severity (*S* ∈ 0: minor, 1: mild, 2: moderate, and 3: severe) was compared against the one from the clinical assessment and the average accuracy for all the subjects was considered as the method’s performance.

**Algorithm 2** Predict Degree of Motor Fluctuation Severity**Input:** Distribution of degree of change in UPDRS III score: $\left\{d_{0},d_{1},d_{2},d_{3}\right\}$
Initialize $p_{0},p_{1},p_{2},p_{3}$ to 0.**if**$\frac{\sum_{i=1}^{3}d_{i}}{d_{0}}>0.3$**then**set $p_{0}$ to N/A**for**s=1 to 3**repeat**$p_{s}=\frac{d_{s}}{\sum_{s=0}^{3}d_{s}}$**End for****else if**$\frac{\sum_{i=1}^{3}d_{i}}{d_{0}}\leq 0.2$**then**set $p_{0}$ to 100%set $p_{1},\;p_{2}$ and $p_{3}$ to N/A**else**S= INCset $p_{0},\;p_{1},\;p_{2}$ and $p_{3}$ to N/A**break****end if**$S=argmax_{s}{\left\{p_{s}\right\}}_{s=0:3}$
**Output:** Predicted degree of motor fluctuation severity (S) and ACUPDRS III percentage {$p_{0},p_{1},p_{2},p_{3}\}$

## 3. Results

**Figure 3:**
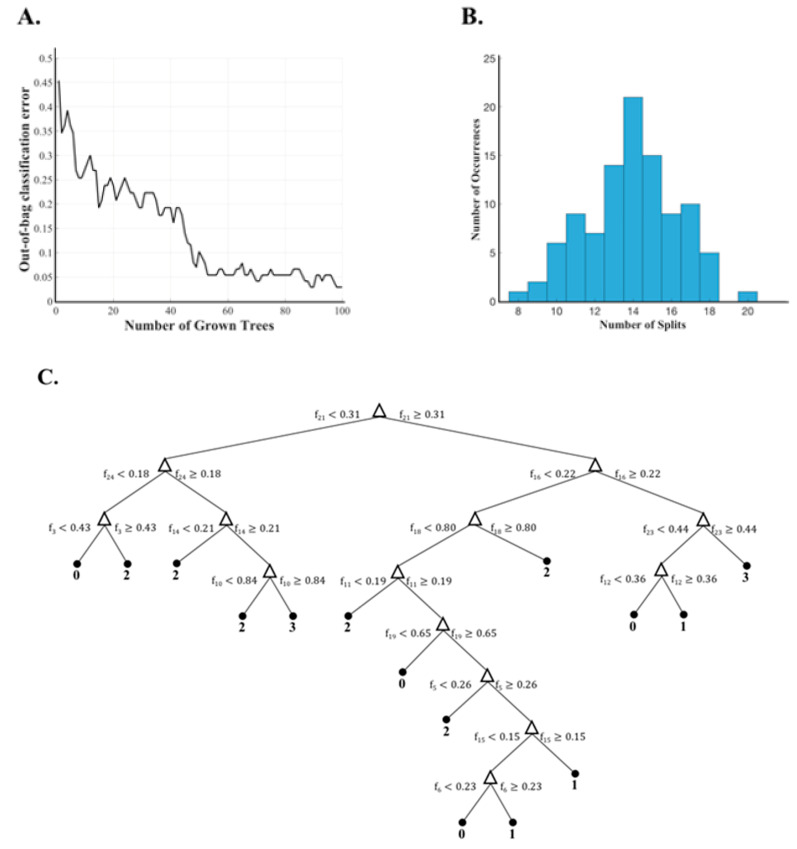
Random Forest decision tree data. (**A**) The change in out-of-bag error as the number of grown trees increased. (**B**) The occurrence of node splits. (**C**) A sample tree with 14 node splits.

The collected data in Section 2.1 was arranged in the form of pairs of rounds with their corresponding sensor and UPDRS III score data as described in Section 2.2. This resulted in a total of 130 pairs of rounds. The described hybrid feature extraction (Section 2.3) was used to map every pair of rounds into the incremental feature space. The extracted features were then modeled into the degree of UPDRS III score change using the classification model explained in Section 2.4. Leave-one-subject-out cross-validation was repeated to evaluate the described method. Figure 3 shows some interesting data about the learned RF-based classifier for one of the repetitions with 24 incremental feature attributes. The out-of-bag classification error decreases with the number of grown trees (Figure 3A) and reaches to a relatively small value after about 60 ensemble decision trees were learned. This observation demonstrates that the number of trees of 100 was an appropriate selection. The occurrence of different number of node splits was calculated and shown in Figure 3B. The number of node splits ranged from 8 to 20. However, most trees had node splits in the range of 1315. In Figure 3C, we showed an example of the learned tree with 14 splits. This tree shows a case where the 21st incremental feature attribute (change of the cluster centers of the two rounds in one pair) resulted in the highest information gain.

**Figure 4:**
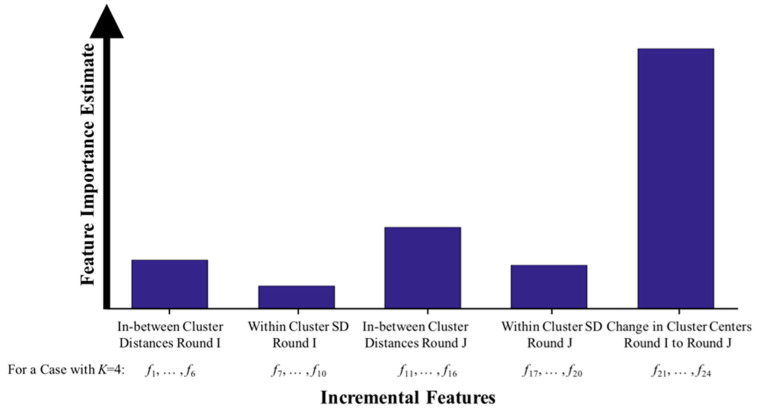
Estimates of features’ importance for five feature categories. The features representing the change in the cluster centroids were the most important features to the trained decision trees.

Another interesting data is presented in Figure 4 where the estimated feature importance to the final decision are shown. The feature importance values were estimated by summing changes in the risk due to splits on every feature value and dividing the sum by the number of branch nodes. In this figure, the extracted incremental features were divided into five groups and their average importance estimate is presented as a bar graph for each group. Please note that the feature attributes that indicate the change in the cluster centroids (i.e., the longitudinal changes in the PD symptom manifestation between the two rounds) provided the highest effect on the trained decision trees, followed by in between centroid distances in Round J and Round I.

**Figure 5:**
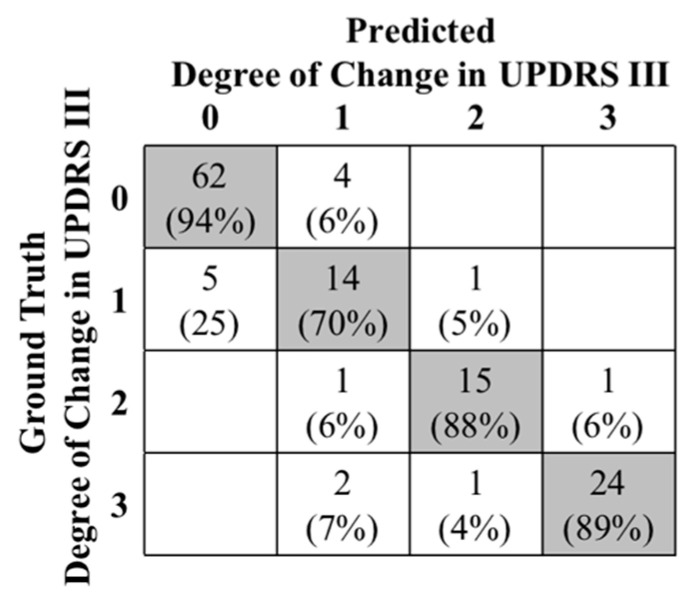
Confusion matrix of the predicted the degree of change in UPDRS III score (0 to 3).

Next, the results of the leave-one-subject-out cross-validation, considering all the 24 subjects, are presented in a confusion matrix of Figure 5. The predicted degree of change in UPDRS III scores (03) of every two rounds were compared to the ground truth values. There was a total of 130 round pairs out of which 115 were correctly classified, resulting in a classification accuracy of 88.46%. The distribution of the misclassified pairs of rounds is shown in the confusion matrix of Figure 5. As shown, the rounds with mild change in UPDRS III score, with an only 70% accuracy, had the most misclassification percentage (25% were classified as minor and 5% as moderate). The reason is that slight changes of motor function patterns in mild motor fluctuation stages can be concealed by the patterns of different activities that the subjects performed during different rounds. As a result, the accurate detection of minor changes in UPDRS III score became more challenging for the algorithm. As a matter of fact, our analysis showed that 86.7% of the misclassified pairs of rounds occurred when the rounds were from different activities. Specifically, we found that this misclassification occurred when only one of the rounds was resting (40%) or walking (26.7%). There was no specific pattern in the remaining 20.0% misclassification.

Finally, we present the results of the predicted degree of motor fluctuation severity in Table 3. For all the 24 subjects in this study, the ACUPDRS III percentages (see Table 3 columns $p_{0},p_{1},p_{2},p_{3}$) and predicted degree of motor fluctuation severity (Table 3 column *S*) were computed as explained in Section 2.6. Considering the 20 subjects who reached their ON state during the data collection process, our algorithm successfully detected the degree of motor fluctuation severity for all the subjects except in two cases (subject #4 and subject #14). Our algorithm correctly resulted in an INC decision for two of the four subjects who did not reach their ON state during the experiments. Hence, the algorithm was successful in 20 out of 24 subjects resulting in an overall classification accuracy of 83.33%.

## 4. Discussion

### 4.1. Efficiency of The Algorithm in Detecting Degree of Motor Fluctuation Severity

A new sensor-based assessment system and algorithm combination was presented that can provide objective and comprehensive measures of motor fluctuation severity. The approach is passive meaning that it provides the assessments as the subjects perform their daily, free-living activities without requiring them to perform any predefined activities. The algorithm extracts a set of incremental features based on a new hybrid feature extraction method and associates the longitudinal changes in the sensor data patterns with the degree of motor fluctuation severity as measured by clinical assessments. The algorithm was tested on data from 24 subjects who performed a broad range of daily life activities, which supports the novelty aspect (2) of our approach as described in the Introduction Section.

Our analysis supports the novelty aspect (1) of our approach, meaning that the algorithm can identify the degree of motor fluctuation severity as assessed from clinical examinations. This high performance was evident by the high leave-one-subject-out classification accuracy (Table 3). Also, the false detected degree-of-severity cases were not too far from the ground truth. The ACUPDRS III score for subject #4 is 16, which is on the boarder of the 2 and 3 severity degrees, and our algorithm detected ACUPDRS III as 2 instead of 3. The same observation was made in the case of subject #14. The ground truth ACUPDRS III is 5 and our algorithm detected it as 1 instead of 0. For two of the four PD subjects who did not convert to their ON state during the experiment (i.e., ACUPDRS III of INC), the algorithm estimated a degree of ACUPDRS severity of other than INC. In the case of subject #22, a severity degree of 0 was detected and a severity degree of 1 was detected for subject #24. This observation indicates that the identification of INC cases is challenging for our algorithm. This aspect of the algorithm could be improved in a long-term monitoring application, where the algorithm can be applied on a longer duration of the data so that both the ON and OFF states be present in the analysis interval. Another observation was that there was no significance dependency on the activities for differentiating between severity degrees of 0, 2, and 3 (see Figure 5). Only in case of severity degree of 1 we observed as association between misclassified cases and activity varieties. Differentiation of a small change in the severity degree is more challenging to the algorithm as the subject performs different types of activities. However, as evident by the high classification accuracy (Table 3), this dependency was reduced by the decision-making process of Algorithm 2 that uses a collective distribution of the detected UPDRS III changes to detect the subjects’ severity degree. Hence, even with 30% of misclassification of severity degrees of 1, the algorithm detected the correct motor fluctuation severity degree in 83.33% of the subjects.

### 4.2. Comparison to Other Studies

Our algorithm can detect the degree of motor fluctuation severity (i.e., ACUPDRS III) without using the medication ON and OFF state information of the patient. The trained model is applied only on the sensor data and can detect ACUPDRS III severity score from the longitudinal analysis of the sensor data without accessing the medication ON and OFF states of the patient. However, all the existing approaches attempt to estimate UPDRS III scores for a given data interval and the information about the medication ON and OFF states must be given to provide the clinical actionable ACUPDRS III severity score between the two states. For example, the work in Ref. [35] attempts to identify UPDRS scores using smartphone data while the subjects performed 5 tasks (voice, finger tapping, gait, balance, and reaction time) on a smartphone application; however, such approaches have to rely on another algorithm to detect the subjects’ medication ON and OFF states in order to provide the actionable information that is needed for medication adjustments of troublesome motor fluctuation in PD subjects. Our algorithm can provide clinically actionable information to adjust medication states independent on a medication ON and OFF detection algorithm.

Moreover, our algorithm detects changes in motor fluctuation severity regardless of the patterns of the concurring activity. This is while the current UPDRS III score detection methods mainly require the subjects to perform some specific activities, such as leg agility task [16]; finger tap and hand grasp actions used in the UPDRS examination [17]; leg agility, sit–stand, and gait tasks [18]; handwriting, speech, and gait [19]; finger tapping, diadochokinesia, and toe tapping [9]; and finger tapping, speech, and gait [20,35]. These approaches are not well suited for the medication adjustment application as they require the subjects’ to engage with the system continuously and actively to provide comprehensive assessment of the temporal variability of motor fluctuation. A recent work in Ref. [26] attempts to estimate severity of UPDRS scores as subjects perform their daily living activities; however, they rely on medication ON and OFF estimation for their detection, which could add another level of error and complexity to the estimation. The reason for our success can be explained by the nature of our hybrid feature extraction method, which enables the classifier to associate different motor fluctuation severity scores with the longitudinal changes in the PD manifestation on the sensor data instead of the PD patterns in each individual segment that can be distorted by the activity patterns. This justification can be further supported by the data in Figure 4 (as well as the data in Supplement Figure S1), which indicates that the features representing the changes in the clusters of the symptom-based features had the most importance to the classifier’s decision. This supports the novelty aspect (3) of our proposed algorithm meaning that an incremental feature extraction approach as developed in our work is more effective than the conventional approaches, which attempt to individually model each segment of data [14,15,16,17,18,19,20,21,22,23,26].

### 4.3. Clinical Implications

Our sensor-based passive assessment system and algorithm combination has the potential to significantly improve the success of PD care. It will enable the development of an in-home monitoring system that provides comprehensive, clinically actionable information about a PD subject’s motor fluctuation severity (i.e., degree of ACUPDRS III severity). Because this sensor information correlates with the clinician-measured degree of ACUPDRS III severity, it could serve as a proxy for clinical measurement that could be applied in telehealth applications. An example clinicalimplication of the estimations from our algorithm, as described in the novelty point (3) of the Introduction Section, is as follows. A minimal fluctuation could suggest that there is no need for any clinical visits or medication adjustments. In the case of a mild or moderate fluctuation, an appropriate therapeutic adjustment to the dose and/or frequency of the medication could be performed without any onsite clinical assessments. Finally, in the case of a severe motor fluctuation, the patient needs a clinical visit for further evaluations before a change to medication can be performed. Hence, the information provided by our algorithm could signal the need for a therapy adjustment without requiring an in person or ON/OFF clinical evaluation and eliminates the confound of patients’ recall bias, thereby improving healthcare access and delivery and quality of life for the millions of patients afflicted by this debilitating neurodegenerative disorder.

### 4.4. Limitations and Future Work

Our algorithm is evaluated using data from 24 PD subjects as they performed different free-range activities in a clinical setting and the results indicates its significant potential in passive detection of motor fluctuation severity during subjects’ routine daily life free body movements. However, performance of the algorithm for large cohort of PD subjects and in the home setting scenarios with very complex activities warrants further investigations. In this work, we developed an ad hoc, histogram-based Algorithm 2 to estimate motor fluctuation severity from the detected degree of change in UPDRS III scores using a hybrid feature extraction and RF-based classifier on the entire available duration of the data from each subject. In a long-term monitoring application, the algorithms can be applied on every 2 h of data with one-hour overlap. Further work will need to be done to determine the appropriate duration of the data and overlap as well as the parameters for Algorithm 2 such that meaningful clinical improvements are achieved following therapy adjustments that were triggered by the algorithm.

## 5. Conclusions

Our objective in this study was to score degree of medication fluctuation severity of subjects with PD using the data from two wearable sensors placed on the upper and lower extremity during free body movements. To do this, we used a hybrid feature extraction approach that consisted of a traditional and a new incremental feature extraction method. The former extracted symptom-based features from different segments of the sensor data and the latter extracted features representing the longitudinal changes in the PD manifestations. We used the extracted incremental features as inputs to a RF classifier and estimated the degree of change in UPDRS III scores between two segments of data. The results of the developed RF classifier on a long duration of data (24 h) from a subject were used to estimate the degree of motor fluctuation severity using an ad hoc, histogram-based algorithm. Our analysis showed that the developed algorithms can identify the degree of motor fluctuation severity as assessed from clinical examinations. We found that the incremental features representing the longitudinal changes are more effective, in detecting severity scores of the motor fluctuation regardless of the patterns of the concurring activity, than the traditional features representing each segment of the data. The developed hybrid feature extraction and RF classification algorithm provides a reliable approach for estimation of the degree of change in UPDRS III scores between two segments of data. However, further investigation is needed to adjust the parameters of the histogram-based algorithm for long-term monitoring applications.

## Figures and Tables

**Table 1:** Participants’ Characteristics.

Patient Characteristics	
Number of Subjects (M,F)	24 (14,10)
Age (years)	58.9° ± 9.3
Disease Duration (years)	9.9° ± 3.7
Levodopa Equivalent Dose (mg)	1251° ± 468
OFF State Hoehn & Yahr	2.1° ± 0.4
OFF State UPDRS Part III	29.7 ± 12.3
AC-UPDRS Part III *	14.4 ± 8.2

* For the 20 Subjects who converted to their OFF state.

**Table 2:** Extracted features representing bradykinesia and tremor symptoms. The features that represent both symptoms are shown in separate rows. All the features were extracted from the time domain except for the shaded features that were extracted from the spectral domain. All the features except for the cross correlation features were extracted from each X, Y, and Z signal separately resulting in three extracted features. The number of extracted features are provided in the last column.

Symptom	Features	#
Bradykinesia	Average Jerk	3
	Peak-Peak Amplitude	3
	Mean Amplitude	3
	SD Amplitude	3
	Skewness	3
	Kurtosis	3
	Sample Entropy	3
	Shannon Entropy	3
	Gini Index	3
	1–4 Hz Signal Power	3
Tremor	First Autocorrelation Peak Amplitude	3
	First Autocorrelation Peak Lag	3
	Cross Correlation XY	1
	Cross Correlation XZ	1
	Cross Correlation YZ	1
	4–6 Hz Signal Power	3
	Percentage Peak Frequencies >4 Hz	3
	Second Spectral Peak Frequency	3
	Second Spectral Peak Power	3
Bradykinesia & Tremor	Autocorrelation Sum	3
	0.5–15 Hz Signal Power	3
	Spectral Entropy	3
	First Spectral Peak Frequency	3
	First Spectral Peak Power	3
Number of Extracted Symptom-Based Features		66

**Table 3:** Prediction of ACUPDRS III percentages and motor fluctuation severity for 24 subjects. The last four subjects indicate the patients who did not experience an ON state during the experiment resulting in an INC for their degree of ACUPDRS III severity.

Subject #	Pair of Rounds #	*d*_0_	*d*_1_	*d*_2_	*d*_3_	Degree of Motor Fluctuation Severity	
		*p*_0_	*p*_1_	*p*_2_	*p*_3_	Predicted (*S*)	Ground Truth
**1**	15	5	0	2	8	3	3
		N/A	0%	20%	80%		
**2**	10	4	4	2	0	1	1
		N/A	67%	33%	0%		
**3**	15	14	1	0	0	0	0
		100%	N/A	N/A	N/A		
**4**	10	4	0	4	2	2	3
		N/A	0%	67%	33%		
**5**	10	3	2	5	0	2	2
		N/A	29%	71%	0%		
**6**	10	2	6	2	0	1	1
		N/A	75%	25%	0%		
**7**	10	4	0	2	4	3	3
		N/A	0%	33%	67%		
**8**	10	3	7	0	0	1	1
		N/A	100%	0%	0%		
**9**	6	2	1	0	3	3	3
		N/A	25%	0%	75%		
**10**	10	6	1	2	1	2	2
		N/A	25%	50%	25%		
**11**	10	4	0	6	0	2	2
		N/A	0%	100%	0%		
**12**	10	2	2	2	4	3	3
		N/A	25	25	50		
**13**	496	369	28	15	84	3	3
		N/A	22%	12%	66%		
**14**	496	378	101	17	0	1	0
		N/A	86%	14%	0%		
**15**	351	347	3	1	0	0	0
		100%	N/A	N/A	N/A		
**16**	595	593	1	1	0	0	0
		100%	N/A	N/A	N/A		
**17**	561	408	20	38	95	3	3
		N/A	13%	25%	62%		
**18**	351	258	27	66	0	2	2
		N/A	29%	71%	0%		
**19**	528	395	89	44	0	1	1
		N/A	67%	33%	0%		
**20**	465	351	10	31	73	3	3
		N/A	9%	27%	64%		
**21**	276	214	51	8	3	INC	INC
		N/A	N/A	N/A	N/A		
**22**	15	13	2	0	0	0	INC
		100	N/A	N/A	N/A		
**23**	10	8	0	2	0	INC	INC
		N/A	N/A	N/A	N/A		
**24**	10	7	2	1	0	1	INC
		N/A	67%	33%	0%		

## Supplementary Materials

The following are available online at http://www.mdpi.com/1099-4300/21/2/137/s1, Supplement Figure S1: The change in the average leave-one-subject-out cross-validation, with no clustering, as the number of the most important feature attributes grows.
