# Supplementary material for: Hybrid Feature Extraction for Detection of Degree of Motor Fluctuation Severity in Parkinson’s Disease Patients
Source: Entropy (Basel). 2019 Feb 1;21(2):137. doi: 10.3390/e21020137 (PMC7514623; doi:10.3390/e21020137)
Supplement: Supplementary file 1 [file entropy-21-00137-s001.pdf]

Supplementary Online Content for "Hybrid Feature Extraction for Detection of Degree of Motor Fluctuation Severity in Parkinson's Disease Patients"

Symptom-based Feature Extraction with no Clustering

We performed an analysis to investigate the importance of clustering in our proposed hybrid feature extraction. Let's consider two rounds of I and J and their extracted symptom-based feature vectors of $\{v_m\}_{m=1:M_I}$ and $\{v_m\}_{m=1:M_J}$, respectively. M_I and M_J denote the number of extracted symptom-based feature vectors for the two rounds. Instead of applying a clustering method on the symptom-based feature vectors to represent the changes in the PD symptoms of the two rounds, we performed the following: First, we calculated the average of each rounds' symptom-based feature vectors (i.e., $\sum_{m=1}^{M_I} v_m$ and $\sum_{m=1}^{M_J} v_m$) and then represented the two rounds as one feature vector by concatenating the two average vectors. This strategy provides a feature vector with 132 (=2x66) attributes. The extracted feature vectors along with their corresponding degrees of change in their UPDRS III were used to train a RF classifier.
Before training the RF classifier, we used the importance score of the RF classifier for each feature to select the feature attributes for the classification of the degrees of UPDRS III changes between two rounds. We used the method that was described in Ref. [1]. An RF classifier with 10,000 trees was trained, and each features' importance were estimated. The features were sorted from the highest to the lowest importance. Next, the sorted features were used in an iterative process to train an RF classifier. The process started with the most important feature and increased the feature attributes with one feature at every iteration until all the features were used in the classifier. The leave-one-subject cross validation of the trained RF classifiers at every iteration is shown in **Supplement Figure 1**. As shown in this figure, the best classification performance was 75%, which was achieved with 74 of the feature attributes. Note that the developed incremental feature extraction algorithm with a clustering method achieved a classification performance of 88.46%, which is 13.46% higher than the method with no clustering.

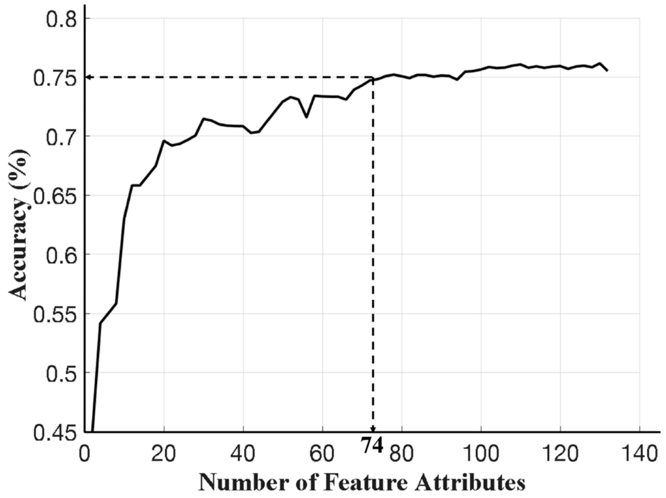

Supplement Figure 1 - The change in the average leave-one-subject-out cross-validation, with no clustering, as the number of the most important feature attributes grows.

[1] Breiman, L., 2001. Random forests. *Machine learning*, *45*(1), pp.5-32.
